# Mitigation of Membrane Fouling in Lignin Recovery from Black Liquor via Surface-Patterned Ceramic Membrane

**DOI:** 10.3390/polym17101424

**Published:** 2025-05-21

**Authors:** Weikang Wang, Ning Kuang, Wenjie Zhao, Qingdang Li

**Affiliations:** 1 College of Electromechanical Engineering, Qingdao University of Science and Technology, Qingdao 266061, China; wangweikang116@163.com (W.W.); kuang.ning@mails.qust.edu.cn (N.K.); 2 College of Sino-German Science and Technology, Qingdao University of Science and Technology, Qingdao 266061, China

**Keywords:** lignin, radial-rib membrane, membrane fouling, shear stress

## Abstract

Among the various methods for recovering lignin from black liquor, membrane separation has gained prominence in the paper industry due to its advantages of uniform molecular weight distribution, high recovery rates, and absence of secondary pollution. However, over time, lignin particles tend to deposit and form a cake layer on the membrane surface, leading to membrane fouling and a decline in filtration flux. To address this issue, this study investigates the construction of ceramic membranes with radial rib patterns, and examines the effects of different trans-membrane pressure differences and radial rib patterns on membrane surface shear force and particle deposition. The research findings indicate that at a trans-membrane pressure difference of 0.5 bar and a blade rotation speed of 1000 r/min, the membrane surface experiences the highest shear force. Compared with those without patterns, ceramic membranes with radial rib patterns can more effectively delay the deposition of particles. Furthermore, it was observed that ceramic membranes combining coarse and fine rib patterns exhibit a more pronounced increase in membrane surface shear force.

## 1. Introduction

The pulp and paper industry is a pillar of the global economy, yet the discharge of salt-laden pulping liquors produced during kraft processing has emerged as a significant environmental concern for the sector and its supply chain. These effluents are dominated by suspended lignin and lignin-derived species whose intricate, recalcitrant structures severely impede natural degradation [1,2,3]. Paradoxically, lignin is the second-most abundant natural polymer after cellulose, containing various functional groups, and exhibiting high thermal stability along with inherent antioxidant and UV-resistant properties. Lignin constitutes a high-value renewable resource, and is the only non-petroleum feedstock capable of furnishing sustainable aromatic compounds [4,5,6,7]. Accordingly, the efficient, low-loss extraction of lignin from saline pulping liquors both transforms waste into value-added product and alleviates the ecological burden of pulping operations, delivering substantial environmental and economic benefits [8,9,10,11].

**Figure 1:**
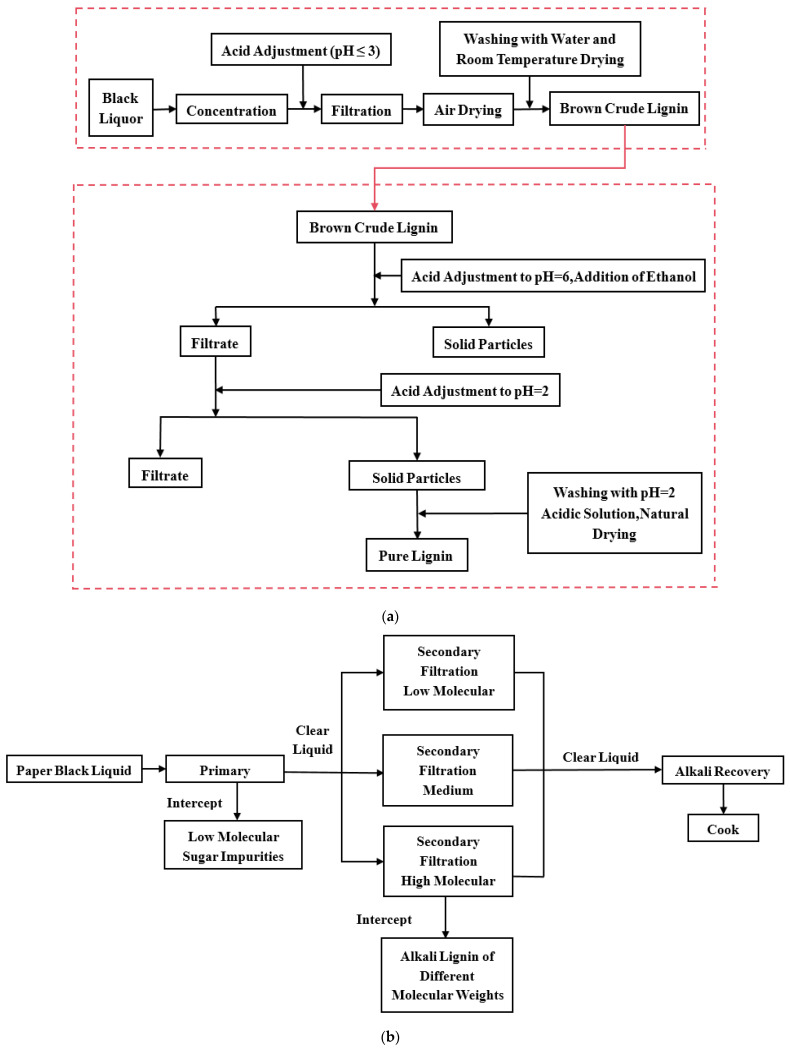
(**a**) Process flowchart for lignin extraction using acid precipitation; (**b**) a schematic of a typical two-stage ultrafiltration membrane separation system.

Currently, lignin is recovered from pulping liquors primarily by acid precipitation or membrane separation (as shown in Figure 1) [12,13,14]. Acid precipitation is simple and inexpensive, but it consumes large amounts of acid and produces secondary pollution, contradicting modern sustainability goals. Membrane separation has therefore gained prominence. Membrane separation methods are distinguished by their low energy consumption, high separation efficiency, and simple system design, which ensures ease of operation and maintenance. Such methods deliver lignin with a narrow molecular weight distribution, high yield, and virtually no secondary waste [15,16,17,18,19,20]. Among the available media, ceramic membranes stand out for their exceptional chemical, thermal, and mechanical stability, making them especially attractive for lignin extraction from kraft liquors [21]. Their broader deployment, however, is curtailed by membrane fouling. Membrane fouling primarily occurs due to the deposition of suspended particles from the feed solution on the membrane surface and within the membrane pores, which reduces permeate flux, elevates operating costs, and lowers product yield [22,23]. Sustained, high-efficiency operation thus depends on strategies that either inhibit fouling deposition during filtration or remove the accumulated layer in situ.

Enhancing hydrodynamic conditions so that high wall shear stresses continuously scour the membrane surface is among the most effective strategies for sustaining permeate flux during filtration [24,25,26]. As a purely physical approach, it avoids both the secondary pollution associated with chemical cleans and the limited service life of consumable reagents. In situ shear-induced scouring provides self-cleaning throughout operation, lowering operating costs, shortening downtime, and ultimately improving separation efficiency. Using CFD simulations of dynamic cross-flow filtration of kraft black liquor, Wenjie Zhao et al. demonstrated that a blade-type rotor generates markedly higher wall shear stress than either disc- or vane-type configurations [27].

Recent investigations demonstrate that micropatterning the membrane surface can substantially enhance anti-fouling performance. Imprinting precise motifs induces periodic, localized flow instabilities that intensify near-wall microturbulence without altering the bulk cross-flow velocity, thereby suppressing fouling deposition [28,29]. Using 3D printing, Zhiyang Lyu et al. fabricated linear grooves on the membrane surface and, through combined CFD simulations and filtration experiments, showed that the patterned membranes generated higher wall shear stress and achieved greater permeate flux than flat controls [30]. Abouther Al Shimmery et al. used 3D printing to emboss a double sine-wave pattern on the membrane surface. Relative to a flat control, the patterned membrane exhibited a higher pure-water flux and, after six cleaning cycles, regained a larger share of its initial permeability [31]. Taken together with other reports [32,33,34,35], these findings reinforce that judicious microstructuring of the membrane surface is a powerful means of elevating flux and mitigating fouling.

In this work, we tackle membrane fouling during lignin recovery from kraft black liquor by imprinting a radial-rib micropattern onto the membrane surface. The ribs induce localized hydrodynamic instabilities that deter lignin particle deposition. We systematically elucidate how trans-membrane pressure, rotor speed, and rib geometry govern wall shear stress, particle accumulation, and flux evolution.

## 2. Numerical Simulation Method

### 2.1. Simulation Theory

Because the process involves no heat transfer, the energy equation is discarded and only the continuity and momentum equations are solved. Turbulence is captured with the RNG variant of the two-equation k–ε model, paired with the standard wall function for near-wall treatment. By explicitly accounting for rotational effects, the RNG k–ε formulation offers superior accuracy in strongly swirling flows. The associated turbulence transport equations are:

(1) $$\frac{\partial (\rho \varepsilon )}{\partial t}+\frac{\partial (\rho v_{j}\varepsilon )}{\partial x_{j}}=\frac{\partial }{\partial x_{j}}(\alpha_{\varepsilon }\mu_{eff}\frac{\partial \varepsilon }{\partial x_{j}})+C_{1\varepsilon }\frac{\varepsilon }{k}(G_{k}+G_{2\varepsilon }G_{b})-C_{1\varepsilon }\rho \frac{\varepsilon^{2}}{k}-R$$

where t is time, ρ is the fluid density, **k** is the turbulent kinetic energy, $x_{j}$ is the coordinate component in the j-direction, $v_{j}$ is the j-direction velocity component, ε is the turbulence dissipation rate, $\alpha_{\varepsilon }$ is the reciprocal of the effective Prandtl number, $\mu_{\mathrm{eff}}$ is the effective (molecular + turbulent) viscosity, $G_{k}$ is the production of k due to mean-velocity gradients, $G_{b}$ is the production of k due to buoyancy, and R represents any additional source term. $\eta =S\frac{k}{\varepsilon }$, $\eta_{0}$ = 4.83, β = 0.012; $C_{\mu }$ = 0.0845, $C_{1\varepsilon }$ = 1.42, $C_{2\varepsilon }$ = 1.68, $\alpha_{k}$ = 1.0, $\alpha_{\varepsilon }$ = 0.769.

### 2.2. Geometry

**Figure 2:**
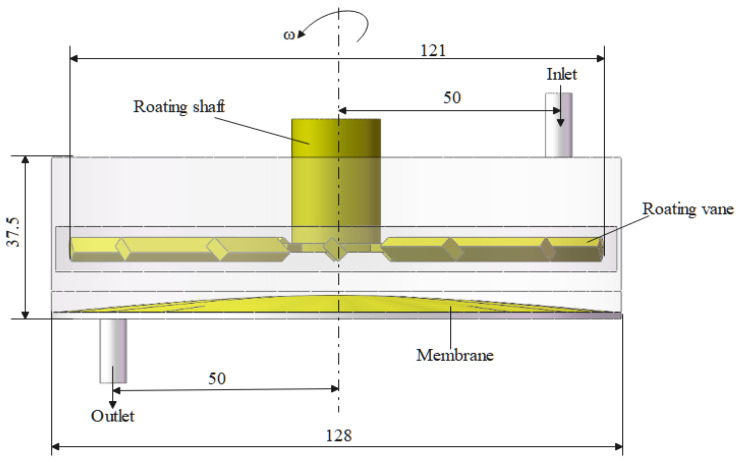
Numerical simulation model of the blade-type dynamic crossflow filtration system.

Figure 2 presents a plan view of the blade-type dynamic cross-flow filtration module fabricated from 304 stainless steel. The filtration chamber has an internal diameter of 128 mm, while the impeller situated immediately beneath the drive shaft measures 122 mm in diameter. Feed and permeate ports are directly integrated into the chamber to introduce black liquor and withdraw the clarified permeate; both ports are deliberately elongated to suppress back-flow. A membrane with an effective area of 3.2 × 10⁻^3^ m^2^ is mounted on the chamber floor and serves as the separation medium.

**Figure 3:**
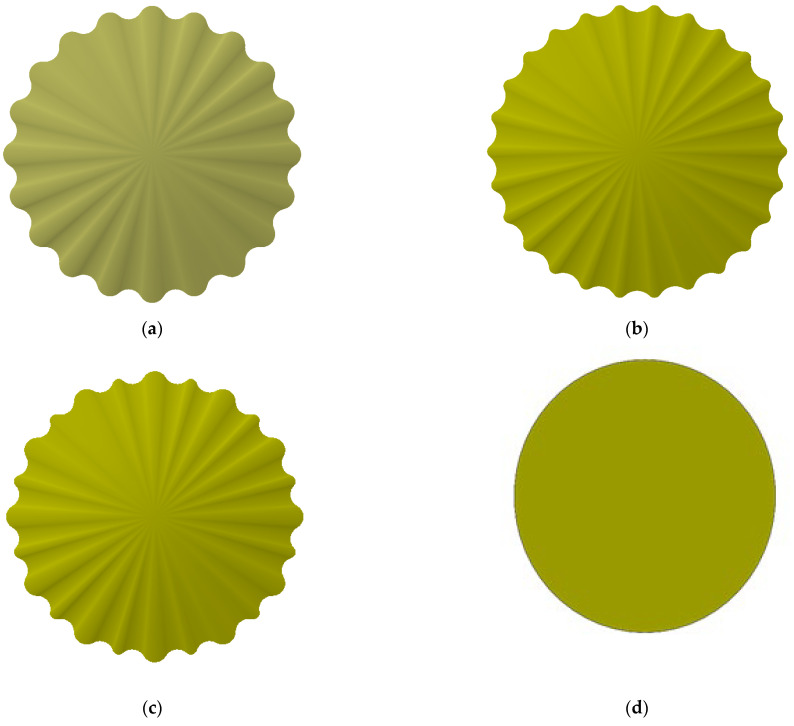
Structural models of four types of ceramic membranes: (**a**) coarse radial-rib membrane; (**b**) fine radial-rib membrane; (**c**) hybrid radial-rib membrane; (**d**) flat membrane.

Four membrane microarchitectures were evaluated in this study: (i) coarse radial ribs, (ii) fine radial ribs, (iii) a hybrid coarse/fine radial-rib pattern, and (iv) a flat, untextured surface. Plan views of these membranes are shown in Figure 3, and their principal characteristics are summarized in Table 1. All membranes were supplied by BOKELA GmbH (Karlsruhe, Germany).

### 2.3. Numerical Simulation

**Figure 4:**
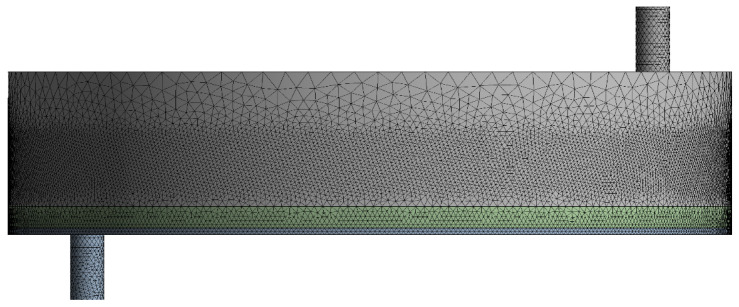
Global mesh distribution of the computational domain.

Numerical simulations of the internal flow field were carried out with a commercial CFD solver. The geometry was preprocessed in SpaceClaim2023 R1, and the computational mesh was generated in ANSYS Meshing2023 R1. As depicted in Figure 4, the final grid comprised roughly 1.55 million cells, and its quality satisfied all solver criteria.

**Figure 5:**
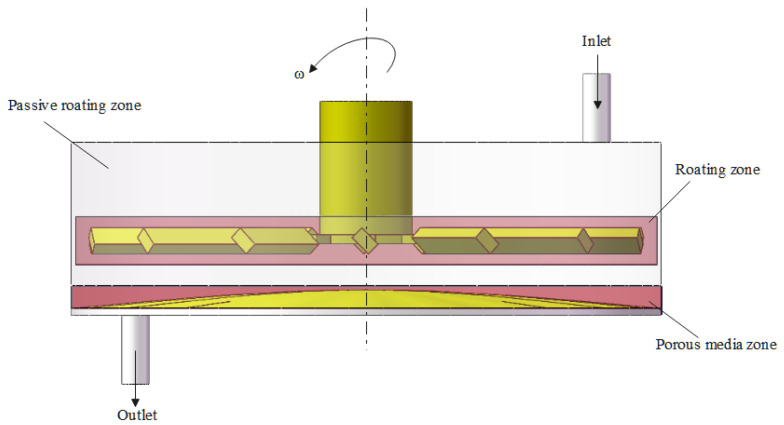
Definition of geometric boundary conditions.

The fluid domain was subdivided into three zones (Figure 5): (i) a rotating reference frame surrounding the impeller, (ii) a porous-medium region representing the membrane, and (iii) a stationary region occupying the remainder of the volume. Owing to the complex blade geometry and steep local velocity gradients, the mesh in the rotating zone was locally refined. A mesh-independence study based on velocity-gradient profiles showed that further refinement altered the results by less than 3%, confirming grid convergence.

A pressure inlet and a pressure outlet were imposed as boundary conditions. Pressure–velocity coupling was handled with the SIMPLE algorithm, and gradient reconstruction employed the least-squares cell-based scheme to accommodate the predominantly tetrahedral mesh. Convergence was declared when both continuity and velocity residuals fell below 1 × 10^−6^.

### 2.4. Settings for the Geometric Boundary Type

The impeller shaft was aligned with the vertical (+z) axis, and the blades rotated counter-clockwise by the right-hand rule. Rotor speeds of 100, 300, 500, 700, 900, and 1000 rpm were simulated. Pressure, velocity, and other field variables were exchanged through internal interfaces linking the rotating, stationary, and porous zones. Trans-membrane pressure differentials of 0.5, 1.0, 1.5, and 2.0 bar were imposed between the feed and permeate sides. The membrane was modeled as a porous medium using Fluent’s built-in formulation, with viscous and inertial resistance coefficients and porosity specified from the NP010 data sheet.

## 3. Simulation Results and Analysis

### 3.1. Velocity Distribution over the Membrane Surface

**Figure 6:**
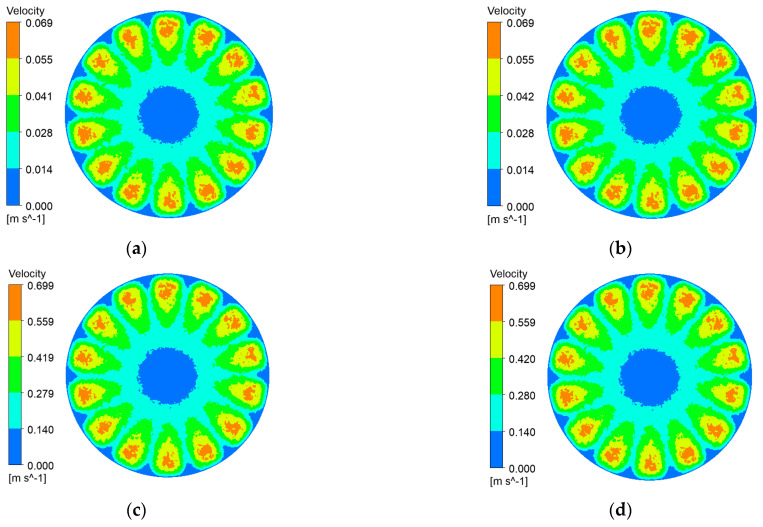
Membrane-surface velocity contours for the coarse radial-rib membrane at various impeller speeds and trans-membrane pressure differences. (**a**) r = 100 r/min, ΔP = 0.5 bar; (**b**) r = 100 r/min, ΔP = 2 bar; (**c**) r = 1000 r/min, ΔP = 0.5 bar; (**d**) r = 1000 r/min, ΔP = 2 bar.

**Figure 7:**
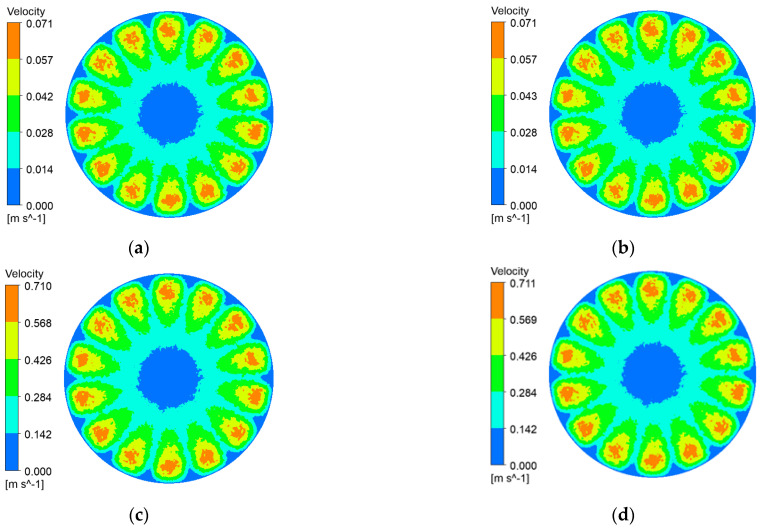
Membrane-surface velocity contours of the fine radial-rib membrane at various impeller speeds and trans-membrane pressure differences. (**a**) r = 100 r/min, ΔP = 0.5 bar; (**b**) r = 100 r/min, ΔP = 2 bar; (**c**) r = 1000 r/min, ΔP = 0.5 bar; (**d**) r = 1000 r/min, ΔP = 2 bar.

**Figure 8:**
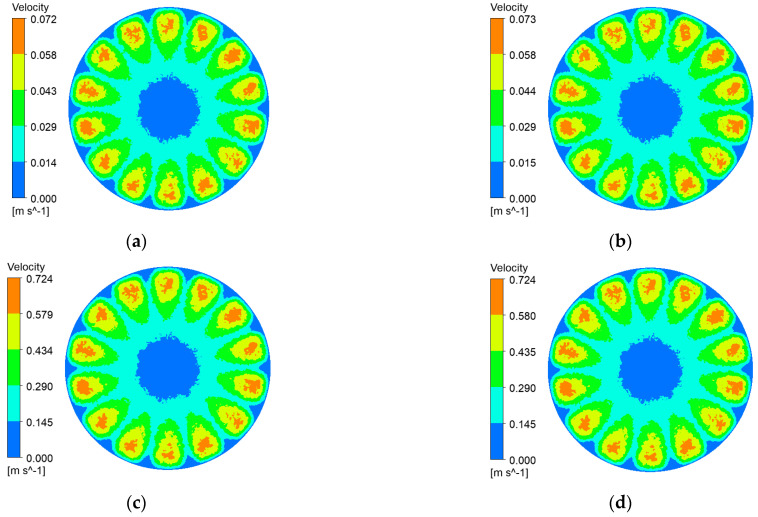
Membrane-surface velocity contours for the hybrid coarse–fine radial-rib membrane at various impeller speeds and trans-membrane pressure differences. (**a**) r = 100 r/min, ΔP = 0.5 bar; (**b**) r = 100 r/min, ΔP = 2 bar; (**c**) r = 1000 r/min, ΔP = 0.5 bar; (**d**) r = 1000 r/min, ΔP = 2 bar.

**Figure 9:**
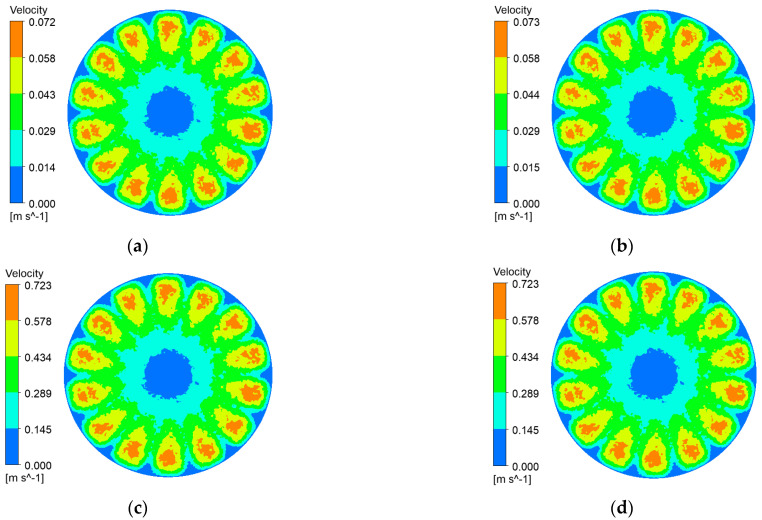
Membrane-surface velocity contours of the flat membrane at various impeller speeds and trans-membrane pressure differences. (**a**) r = 100 r/min, ΔP = 0.5 bar; (**b**) r = 100 r/min, ΔP = 2 bar; (**c**) r = 1000 r/min, ΔP = 0.5 bar; (**d**) r = 1000 r/min, ΔP = 2 bar.

Impeller rotation reshapes the flow regime of black liquor, imparting bulk shear that reaches the membrane surface. The resulting tangential stress curbs lignin-particle deposition, delays cake build-up, and thus helps the ceramic membrane maintain a high permeate flux. Figure 6, Figure 7, Figure 8 and Figure 9 depict membrane-surface velocity fields for the four membrane topographies at impeller speeds of 100 rpm and 1000 rpm, under trans-membrane pressure (TMP) differences of 0.5 bar and 2.0 bar. These two speeds bracket the operating range and therefore capture the limiting shear conditions of interest.

It can be seen from Figure 6, Figure 7, Figure 8 and Figure 9 that, with a fixed impeller speed, the four membranes exhibit only minor variations in surface shear velocity. The effect of speed itself is pronounced: regardless of TMP, the mean surface velocity rises from ≈ 0.07 m s⁻^1^ at 100 rpm to ≈ 0.7 m s⁻^1^ at 1000 rpm—an order-of-magnitude increase.

### 3.2. Shear Stress Distribution Across the Membrane Surface

**Figure 10:**
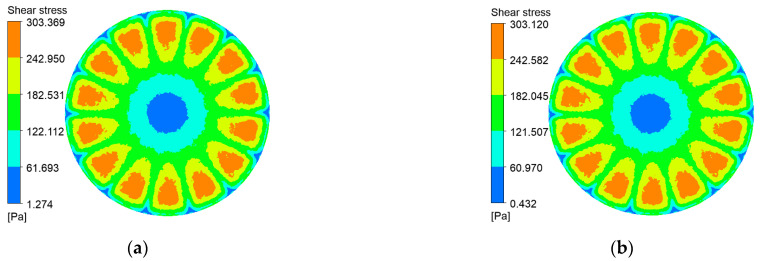

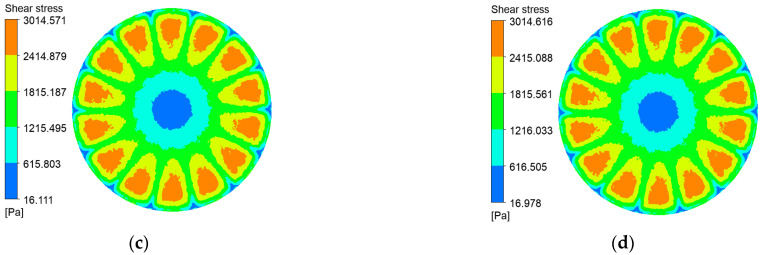
Shear stress contours on the coarse radial-rib membrane surface at various impeller speeds and trans-membrane pressure differentials. (**a**) r = 100 r/min, ΔP = 0.5 bar; (**b**) r = 100 r/min, ΔP = 2 bar; (**c**) r = 1000 r/min, ΔP = 0.5 bar; (**d**) r = 1000 r/min, ΔP = 2 bar.

**Figure 11:**
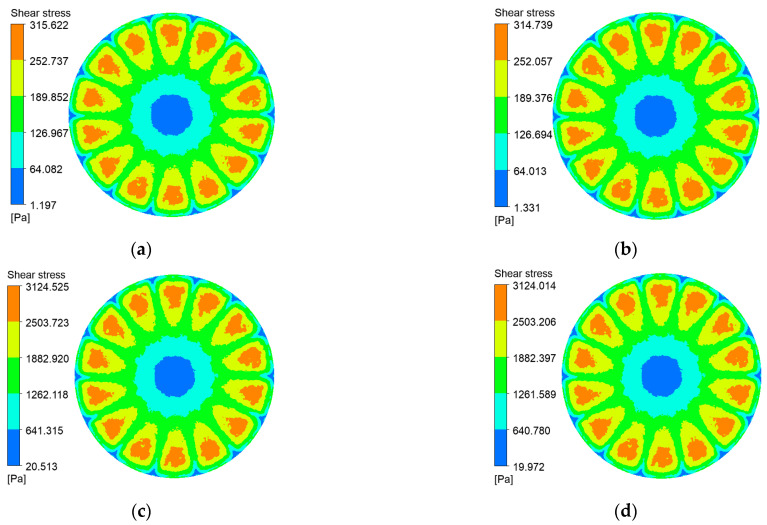
Shear stress contours on the fine radial-rib membrane surface at various impeller speeds and trans-membrane pressure differentials. (**a**) r = 100 r/min, ΔP = 0.5 bar; (**b**) r = 100 r/min, ΔP = 2 bar; (**c**) r = 1000 r/min, ΔP = 0.5 bar; (**d**) r = 1000 r/min, ΔP = 2 bar.

**Figure 12:**
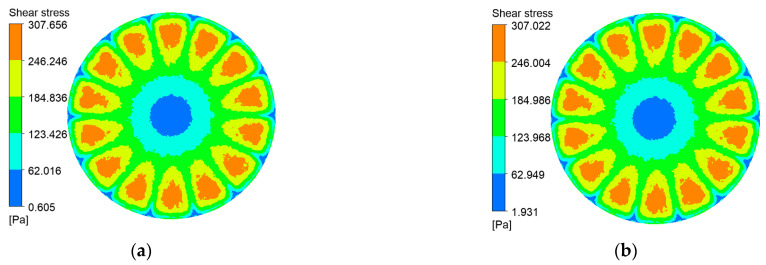

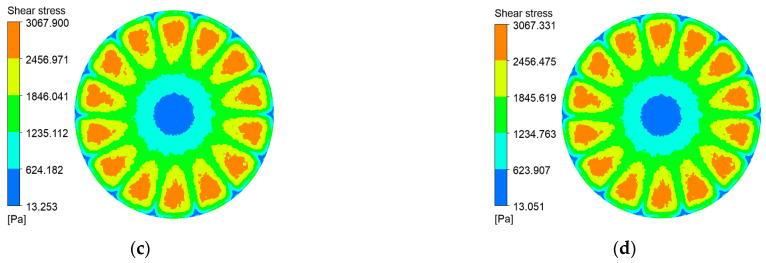
Shear stress contours on the hybrid coarse–fine radial-rib membrane surface at various impeller speeds and trans-membrane pressure differentials. (**a**) r = 100 r/min, ΔP = 0.5 bar; (**b**) r = 100 r/min, ΔP = 2 bar; (**c**) r = 1000 r/min, ΔP = 0.5 bar; (**d**) r = 1000 r/min, ΔP = 2 bar.

**Figure 13:**
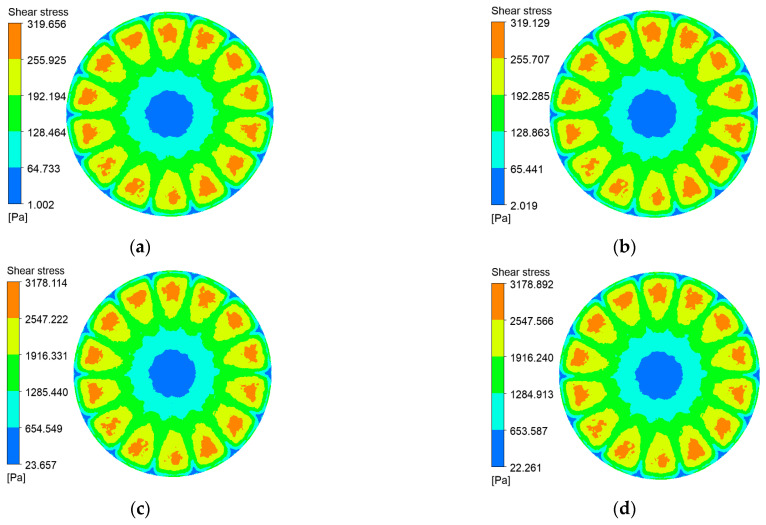
Shear stress contours on the plane membrane surface at various impeller speeds and trans-membrane pressure differentials. (**a**) r = 100 r/min, ΔP = 0.5 bar; (**b**) r = 100 r/min, ΔP = 2 bar; (**c**) r = 1000 r/min, ΔP = 0.5 bar; (**d**) r = 1000 r/min, ΔP = 2 bar.

Shear stress at the membrane surface is a critical determinant of particle deposition. Figure 10, Figure 11, Figure 12 and Figure 13 present shear-stress maps for three microtextured ceramic membranes and one smooth planar membrane over a range of trans-membrane pressure (TMP) drops and impeller speeds. Although the spatial distributions are broadly similar, the stress magnitudes differ markedly. The highest stresses occur in the vicinity of the rotating blades. At 1000 rpm and TMPs of 0.5 bar and 2 bar, the planar membrane and the fine radial-rib membrane record peak surface stresses of 3124 Pa and 3178 Pa, respectively. By comparison, the hybrid coarse–fine rib membrane reaches a slightly lower maximum of 3067 Pa, yet it exposes the largest area of the membrane to high shear.

### 3.3. Influence of Trans-Membrane Pressure and Impeller Speed on Velocity and Shear Stress at the Membrane Surface

Permeate flux theory points to trans-membrane pressure (TMP) and impeller speed as the chief variables governing shear velocity and shear stress at the membrane surface; the interplay of blade rotation and TMP supplies the driving force that reshapes the near-wall flow. However, the results presented so far in Section 3 demonstrate that surface texturing—most notably the hybrid coarse–fine rib design—offers an equally powerful means of altering local hydrodynamics and elevating surface shear. The sections that follow therefore examine separately the effects of TMP, impeller speed, and membrane patterning on the distribution of shear velocity and shear stress.

#### 3.3.1. Influence of Trans-Membrane Pressure on Velocity at the Membrane Surface

**Figure 14:**
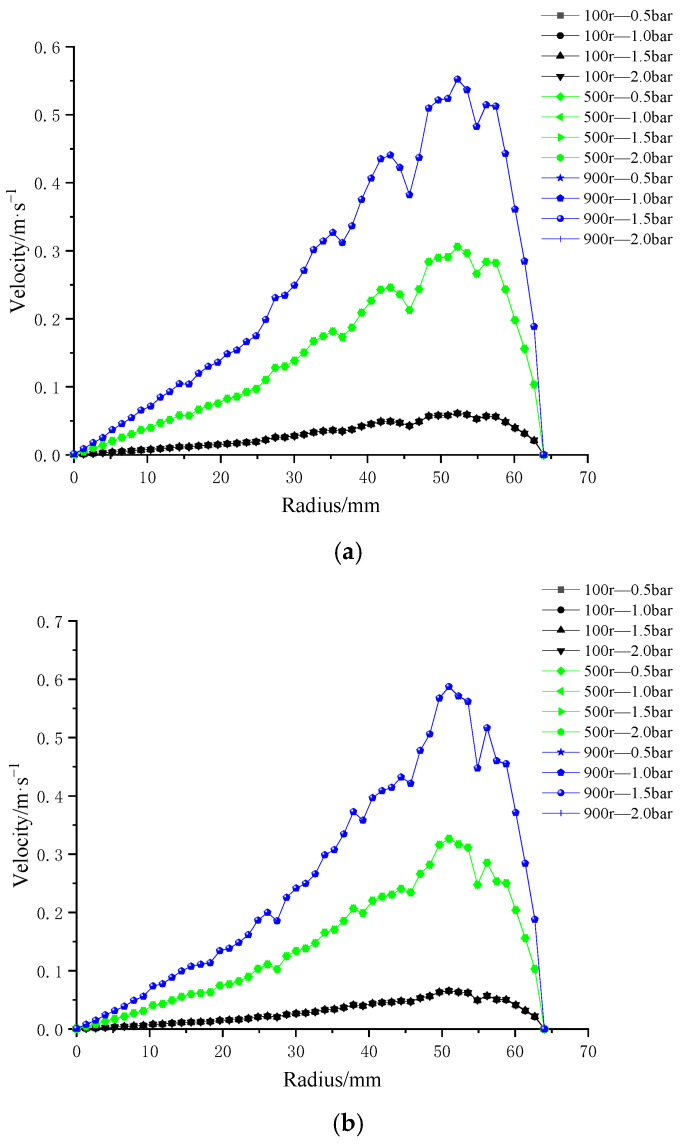

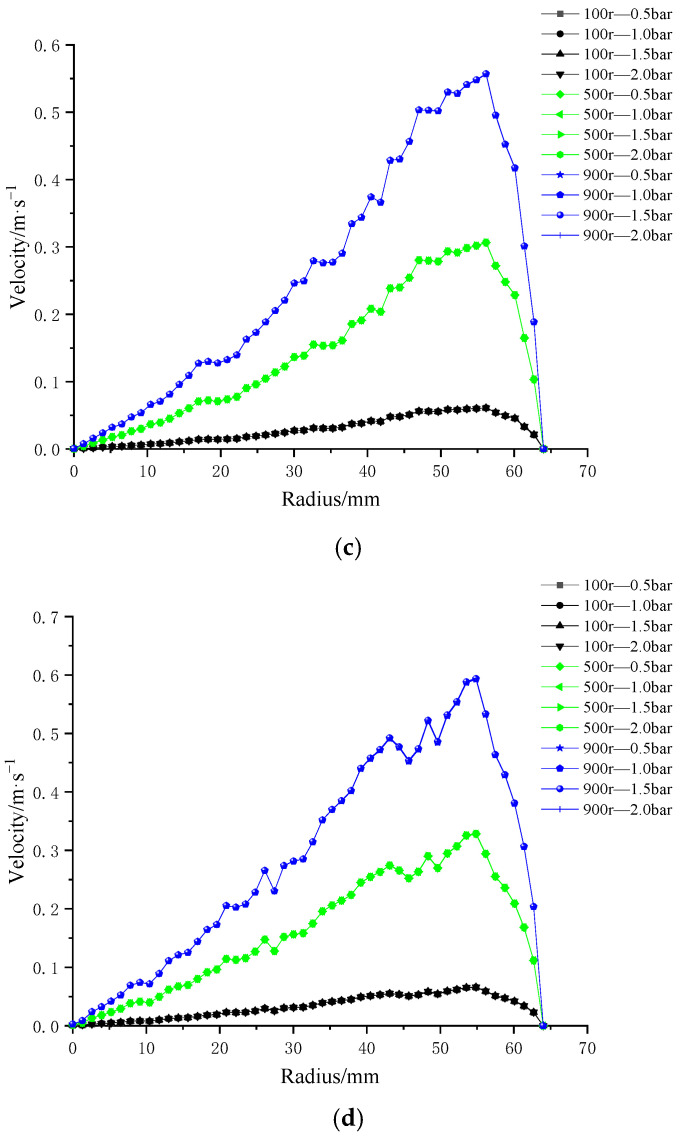
Radial distributions of surface velocity for four ceramic membrane designs at varying impeller speeds and trans-membrane pressure differentials: (**a**) coarse radial-rib; (**b**) fine radial-rib; (**c)** hybrid coarse–fine radial-rib; and (**d**) smooth planar membrane.

To investigate the influence of trans-membrane pressure on membrane-surface shear rate, four types of patterned membrane structures were evaluated under four trans-membrane pressure differences (0.5 bar, 1.0 bar, 1.5 bar, and 2.0 bar) at three rotational speeds (100 r/min, 500 r/min, and 900 r/min), as illustrated in Figure 14. The results revealed that, at a fixed rotational speed, variations in trans-membrane pressure had negligible impact on the shear rate distribution across the membrane surface, with the profiles nearly overlapping. As the membrane radius increased, the shear rate initially rose and then declined sharply, with the turning point corresponding to the outermost edge of the impeller.

Moreover, for all four membrane patterns, the maximum shear rate was consistently observed at the highest rotational speed of 900 r/min. An overall increasing trend in shear rate was evident with rising rotational speed. These findings indicate that impeller rotational speed is the dominant factor governing shear rate at the membrane surface, while trans-membrane pressure plays a comparatively minor role.

#### 3.3.2. Influence of Impeller Speed on Velocity at the Membrane Surface

**Figure 15:**
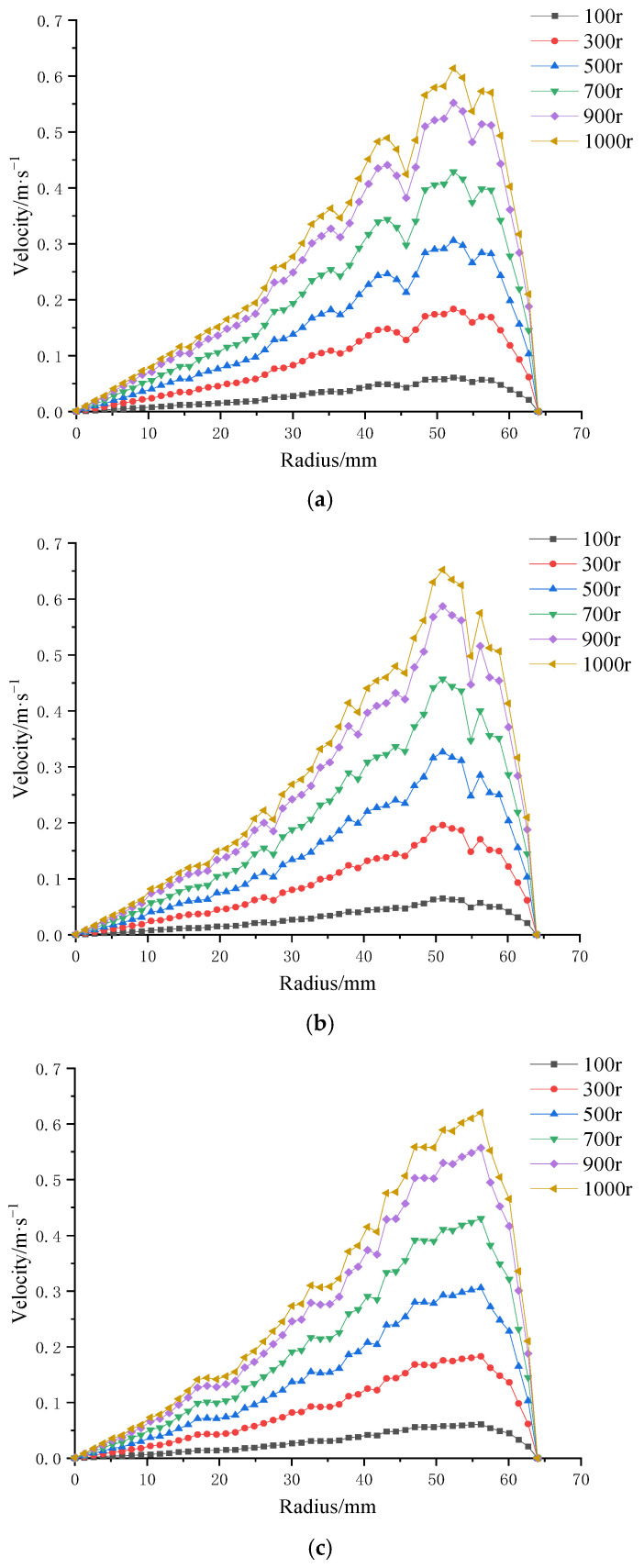

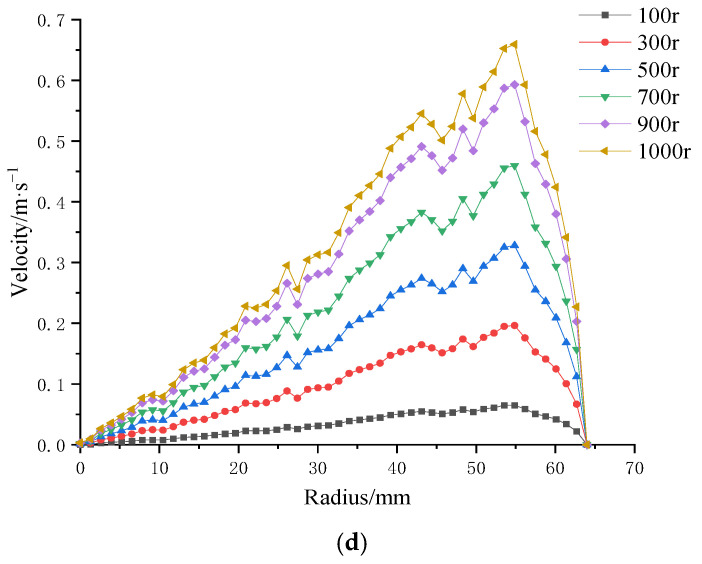
Surface velocity on four ceramic membranes at impeller speeds of 100–1000 rpm under a trans-membrane pressure of 0.5 bar: (**a**) coarse radial-rib membrane; (**b**) fine radial-rib membrane; (**c**) hybrid coarse–fine radial-rib membrane; and (**d**) planar membrane.

Following the analysis of trans-membrane pressure effects, impeller rotational speed was identified as the dominant factor influencing shear rate on the membrane surface. To further investigate this, numerical simulations were conducted to evaluate the shear rate distribution on four distinct patterned membranes under six different impeller speeds (100 r/min, 300 r/min, 500 r/min, 700 r/min, 900 r/min, and 1000 r/min), as illustrated in Figure 15.

The results demonstrate that, for all four membrane patterns, shear rate increases progressively with rising impeller speed. Additionally, shear rate generally increases along the membrane radius. Notably, the membrane with the combined coarse–fine rib pattern (Figure 15c) exhibited a more stable and consistent increase in shear rate with radial distance, without the local decreases observed in the other three patterns. In contrast, the remaining membrane designs showed multiple fluctuations, characterized by alternating decreases and increases in shear rate along the radius.

These findings suggest that the combined coarse–fine rib pattern offers superior performance in enhancing and maintaining high shear rates across the membrane surface, making it a promising design for shear-driven membrane applications.

#### 3.3.3. Influence of Trans-Membrane Pressure on Shear Stress at the Membrane Surface

**Figure 16:**
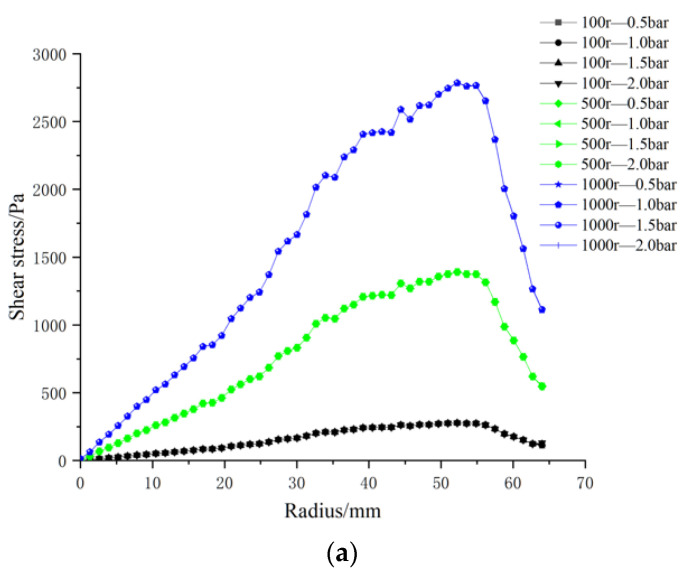

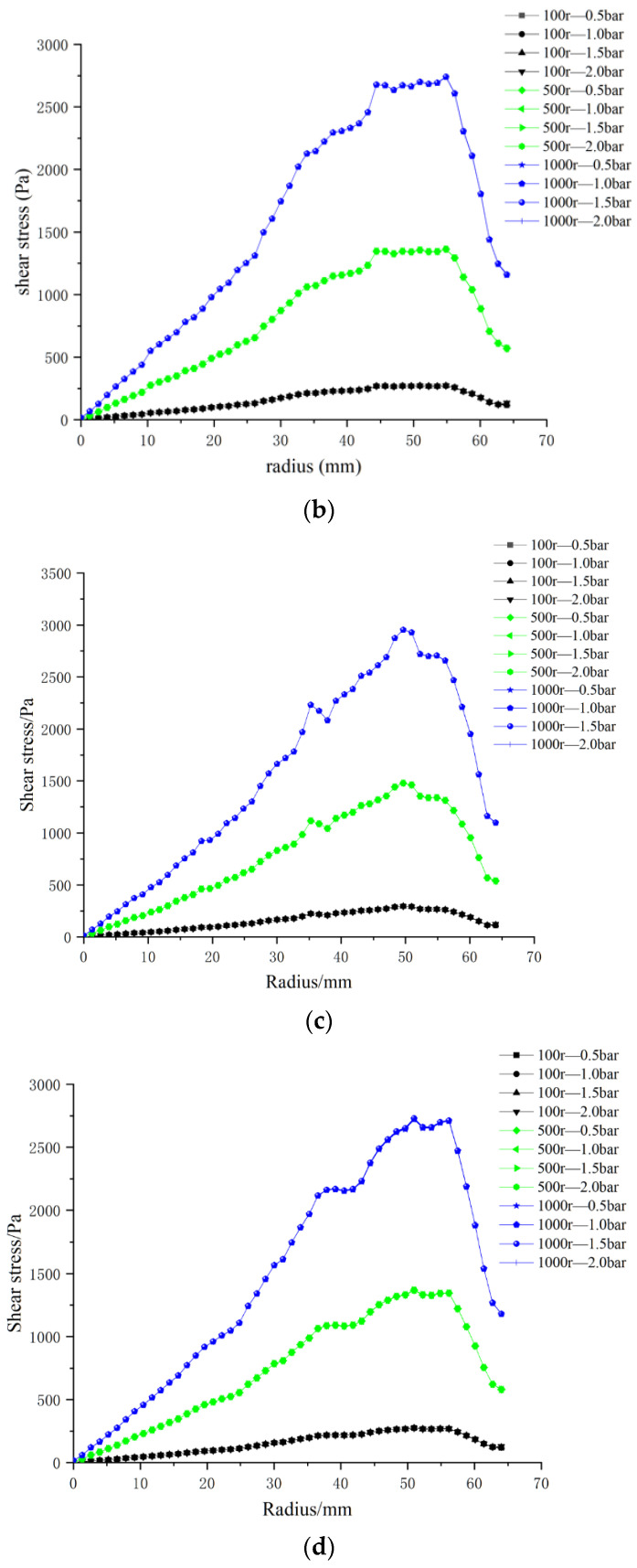
Radial distributions of surface shear stress for four ceramic membrane designs at varying impeller speeds and trans-membrane pressure differentials: (**a**) coarse radial-rib; (**b**) fine radial-rib; (**c)** hybrid coarse–fine radial-rib; and (**d**) smooth planar membrane.

The effect of four trans-membrane pressure (TMP) drops—0.5, 1.0, 1.5, and 2.0 bar—on membrane-surface shear stress was assessed. Figure 16 presents the radial shear-stress profiles obtained at impeller speeds of 100, 500, and 1000 rpm.

At a given impeller speed, the profiles for the four TMPs are virtually superimposed: shear stress rises from the center, attains a maximum at ~50 mm, and then diminishes toward the membrane periphery. By contrast, at a fixed TMP, the stress level is strongly governed by rotational speed, with 1000 rpm producing the highest shear across the entire radius.

#### 3.3.4. Influence of Impeller Speed on Shear Stress at the Membrane Surface

**Figure 17:**
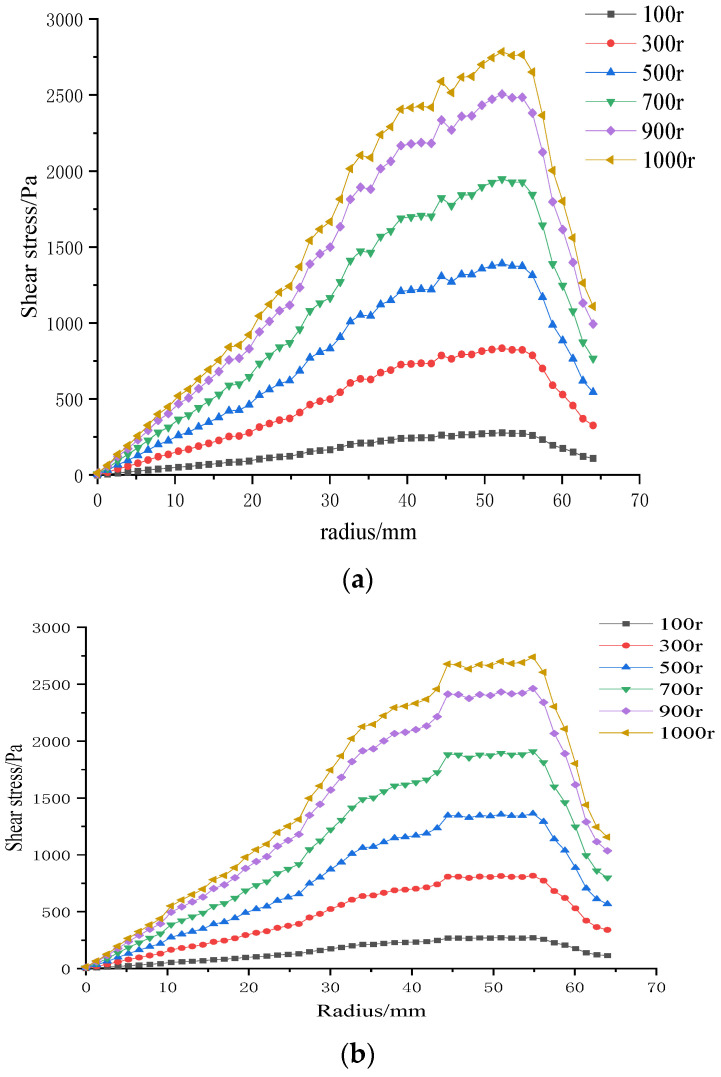

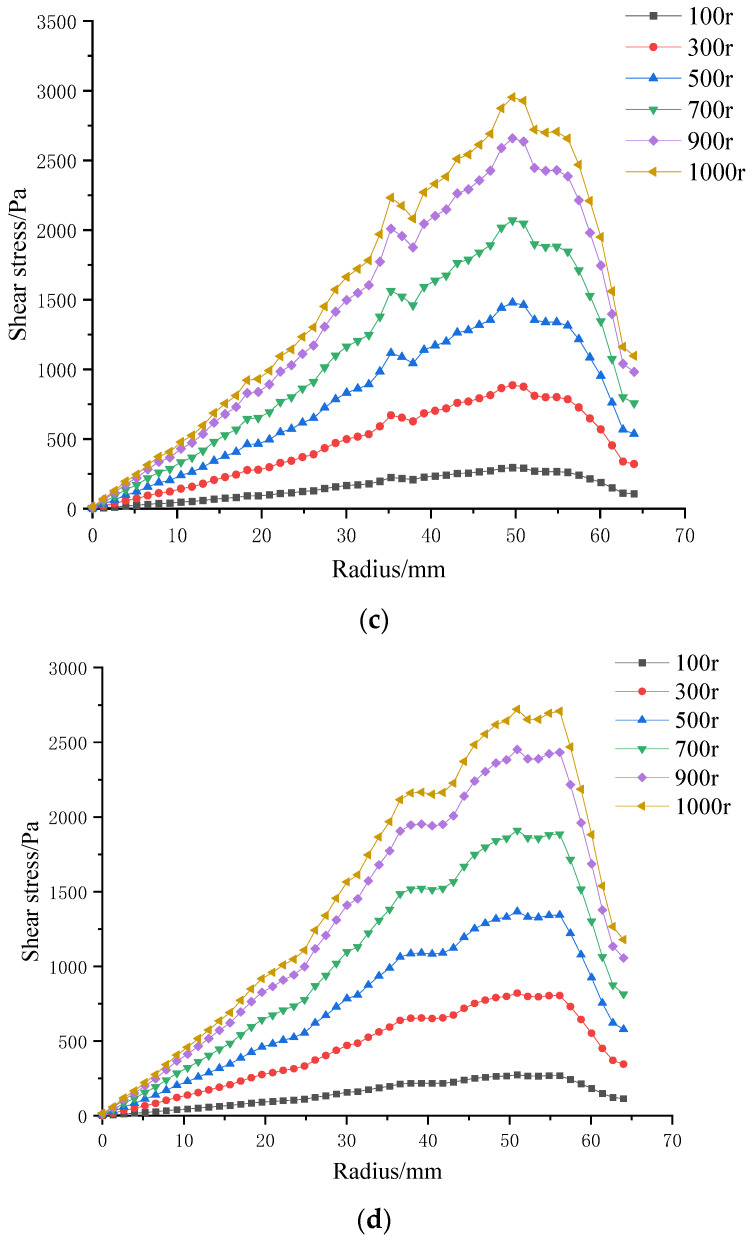
Surface shear stresses on four ceramic membranes at impeller speeds of 100–1000 rpm under a trans-membrane pressure of 0.5 bar: (**a**) coarse radial-rib membrane; (**b**) fine radial-rib membrane; (**c)** hybrid coarse–fine radial-rib membrane; and (**d**) planar membrane.

Section 3.3.3 established that variations in trans-membrane pressure (TMP) have only a minor effect on membrane-surface shear stress, whereas impeller speed is the dominant control variable. Consequently, in the following analysis, TMP is fixed at 0.5 bar, isolating the influence of rotational speed. Figure 17 displays radial shear-stress profiles for three microtextured ceramic membranes and a flat reference membrane across the 100–1000 rpm range.

At constant TMP, surface shear stress rises monotonically with impeller speed for all four membranes. It is lowest at the membrane center, increases with radial distance, and then falls off beyond ≈ 55 mm, where the blade tips approach the chamber wall and the flow encounters heightened wall resistance. The hybrid coarse–fine rib membrane (Figure 17c) is particularly noteworthy: its peak shear stress occurs at a radius of 50 mm, mirroring the extensive high-stress zone seen in the contour plot of Figure 12 (Section 3.2). Among the membranes tested, the combination of blade rotation and hybrid rib topology generates the broadest area of elevated shear on the membrane surface.

### 3.4. Influence of Radial-Rib Surface Patterns on Membrane-Surface Shear Stress

**Figure 18:**
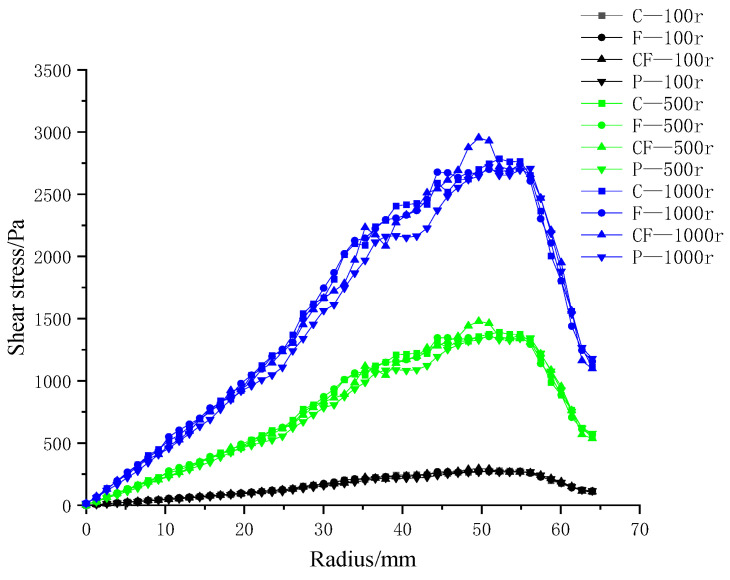
Membrane-surface shear stress distributions for four membrane microstructures at impeller speeds of 100, 500, and 1000 rpm. C—coarse radial-rib; F—fine radial-rib; CF—hybrid coarse–fine radial-rib; P—planar membrane.

This section evaluates the shear-stress field generated on four ceramic membranes. Figure 18 presents the radial distributions at impeller speeds of 100, 500, and 1000 rpm.

At all speeds, the membranes exhibit the same profile: shear stress rises from the center, reaches a peak, and then declines toward the rim. The four curves are nearly coincident at 100 rpm; at 1000 rpm they preserve this shape but separate markedly in magnitude.

For any given speed, each ribbed membrane develops higher shear than the smooth planar reference. Among the ribbed designs, the hybrid coarse–fine pattern delivers the greatest stresses, while the coarse-rib and fine-rib variants are virtually indistinguishable. These results confirm that surface texturing—particularly the hybrid rib architecture—is far more effective than a flat surface in augmenting membrane-surface shear.

### 3.5. Particle Deposition Throughout the Filtration Process

**Figure 19:**
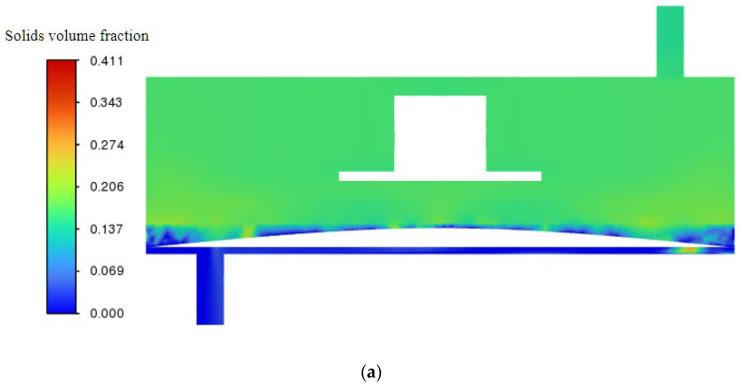

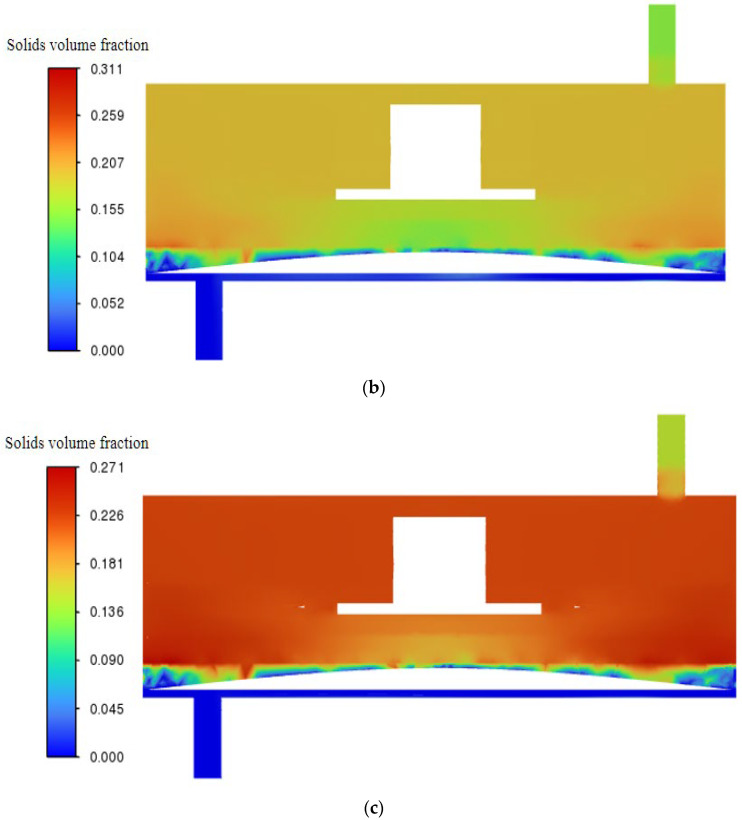
Particle deposition contours for the hybrid coarse–fine radial-rib ceramic membrane at a trans-membrane pressure of 0.5 bar and an impeller speed of 1000 rpm: (**a**) 2 s; (**b**) 20 s; (**c**) 40 s.

Section 3.1 and Section 3.2 present contour maps of surface shear stress and shear velocity for trans-membrane pressure (TMP) differentials of 0.5 bar and 2.0 bar and impeller speeds from 100 to 1000 rpm. The data show that the hybrid coarse–fine rib ceramic membrane operated at 0.5 bar and 1000 rpm delivers the most advantageous combination of surface shear velocity and shear stress. Guided by this finding, a dedicated CFD simulation was performed to track lignin particle deposition on the hybrid membrane during black-liquor filtration (Figure 19). Particle concentration contours are shown at 2, 20, and 40 s; by 40 s, lignin particles occupy roughly 27 % of the chamber volume relative to their initial content in the feed.

## 4. Conclusions

This study demonstrates that the integration of blade-driven rotation with radially ribbed ceramic membrane topographies effectively mitigates flux decline during kraft black-liquor filtration by enhancing near-wall hydrodynamics and suppressing lignin deposition. CFD simulations confirm that impeller speed significantly increases surface shear stress, while trans-membrane pressure has a limited effect. Among the textures evaluated, the hybrid coarse–fine rib design generates the largest high-shear region and most effectively delays particle accumulation, as validated by particle tracking analysis. These findings highlight the potential of combining membrane surface structuring with dynamic shear enhancement as a viable strategy to maintain high permeate flux in the treatment of high-fouling industrial streams.

## Figures and Tables

**Table 1:** Membrane information in the experiment.

Name	Membrane Material	Permeability [LMH/bar]	PH Range	Nominal M.W.C.O (Da)
NP010	PES	≥5	0.0–14.0	1000–1200

## Data Availability

The original contributions presented in this study are included in the article. Further inquiries can be directed to the corresponding authors.
